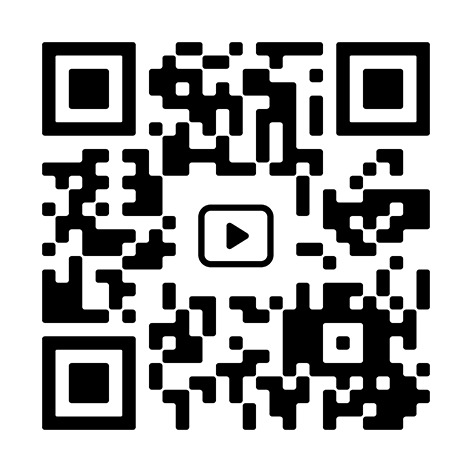# Laser for twin-to-twin transfusion syndrome: a guide for endoscopic surgeons

**Published:** 2020-01-24

**Authors:** L Van Der Veeken, I Couck, J Van Der Merwe, L De Catte, R Devlieger, J Deprest, L Lewi

**Affiliations:** Academic Department of Development and Regeneration, Woman and Child, Biomedical Sciences, KULeuven and Clinical Department of Obstetrics and Gynecology, University Hospitals Leuven, Herestraat 49, 3000, Leuven, Belgium;; Institute for Women’s Health, University College London, London, United Kingdom.

**Keywords:** Fetoscopy, Laser, monochorionic twin pregnancy, twin twin transfusion, TTTS, TOPS

## Abstract

Twin-to-twin-transfusion syndrome (TTTS) is the most important cause of handicap and death in monochorionic twin pregnancies. It is caused by a certain pattern of anastomoses between the two fetal circulations leading to an unbalanced blood and fluid transfer. This leads to severe amniotic fluid discordance and variable degrees of cardiac dysfunction. Untreated, this condition has a very poor survival rate. Fetoscopic laser has been shown to be the best first line treatment, which aims to dichorionise the placenta therefore arresting the inter-twin transfusion. Fetoscopic laser is a causative therapy, which aims to functionally create a dichorionized placenta hence arresting inter-twin transfusion. This is achieved by percutaneous sono-endoscopic coagulation of placental anastomoses. In addition, redundant amniotic fluid is drained. Fetoscopic laser coagulation of chorionic plate anastomoses is safe and effective. There is level I evidence that it is the best treatment modality, in particular when the placental surface is lined along the vascular equator. A recent meta-analysis confirmed an increased fetal survival and decreased risk for neonatal and pediatric neurologic morbidity. Laser therapy is the first line therapy for TTTS. The technique is quite standardized and safe and effective in experienced hands. Herein we describe the technique and current instrumentation used for this procedure.

## Introduction

Monozygotic twins are the result of the cleavage of one fertilized egg. In about 70% of monozygotic twins, cleavage occurs after the 4 th day post fertilization, resulting in a twin set that shares a single placenta, hence monochorionic twins (MC) (Hall, 2003). Although MC twin pregnancies only make up 20% of all twin pregnancies, they have a considerably higher risk for mortality and morbidity than dichorionic (DC) twins. This is due to nearly ever-present exchange of blood through vascular anastomoses between the two fetal circulations. In case of an imbalance in this exchange, it can lead to the development of a severe amniotic fluid discrepancy, better known as twin-to-twin transfusion syndrome (TTTS). This occurs in around 10% of MC pregnancies (Lewi et al., 2010).

**Figure 1:**
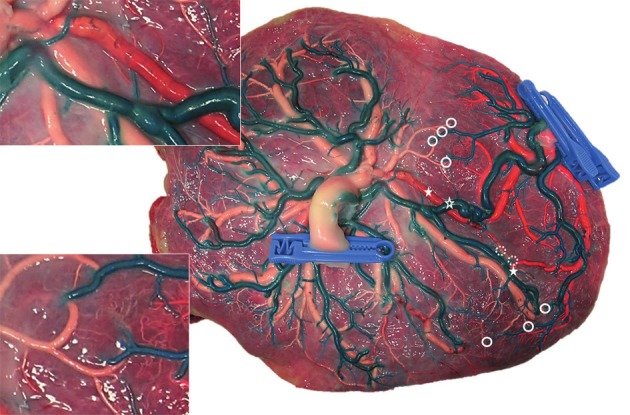
Image from a placenta of a monochorionic pregnancy. Multiple anastomoses are present: 1AA (open star) ; 2 VV (full star) ; multiple AV (dotted circle) ; multiple VA (full circle). Reproduced with permission and copyright: UZ Leuven, Belgium.

**Figure 2:**
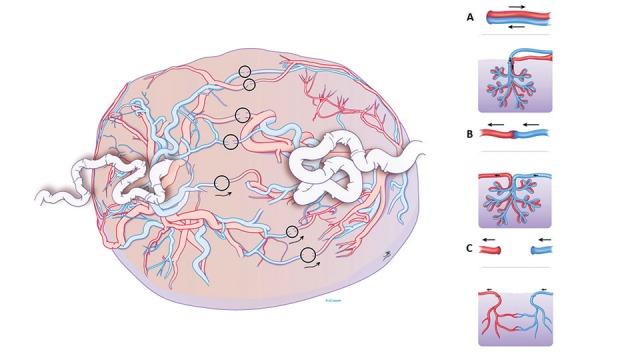
Left: Schematic view of the placenta with the different types of anastomoses. Right: A) normal cotyledon with paired vessels, from and to the same fetus. B) Shared cotyledon, with artery and vein belonging to a different fetus. C) Superficial unpaired vessels, that do not connect closely. Drawing Myrthe Boymans, Reproduced with permission and copyright: UZ Leuven, Belgium.

Twin-to-twin transfusion syndrome corresponds to the specific angioarchitecture of connecting chorionic plate arterio-arterial (AA), veno-venous (VV) and bidirectional arterio-venous (AV) connections (Figure 1 and 2) that will determine whether a net blood and fluid shift from one twin to the other arises (Acosta-Rojas et al., 2007, Lewi et al., 2008). In that case the donor becomes hypovolemic with oliguria, leading to oligo- anhydramnios. On the other hand, the recipient develops hypervolemia leading to polyuric polyhydramnios. Besides a transfusion imbalance, there is hormonal dysfunction and usually a variable degree of unequal placental sharing (Mahieu-Caputo et al., 2001). The condition is typically occurring in mid gestation yet early (<16 weeks) and late manifestations (>26 weeks) are possible.

The presenting ultrasound features are the base for staging TTTS. Currently, the most common system is that proposed by Quintero et al. (1999). Irrespective of the stage, there is severe fluid discrepancy. To qualify for oligohydramnios, the deepest vertical pocket (DVP) should be <2cm. In Europe a cut off for polyhydramnios of >8cm prior to 20 weeks and > 10cm after 20 weeks is used (Senat et al. 2004). In contrast, in the USA the cut-off of 8cm is maintained throughout gestation (Quintero et al. 1999). Before 18 weeks , some authors suggest to use a cut-off of > 6cm to define the polyhydramnios, although this is not yet generally accepted (Lewi, 2010). In stage I disease, there is still bladder filling in the donor. In stage II, bladder filling is absent. In stage III, there is obvious hemodynamic impact evidenced by Doppler abnormalities in the umbilical artery, vein or ductus venosus. In stage IV there is fetal hydrops (Quintero et al. 1999). Stage V means fetal death of one or both twins. Although growth discordance often accompanies TTTS, it is not a diagnostic criterion.

Furthermore, the condition should be differentiated from Twin-Anemia Polycythemia Sequence (TAPS). The latter is caused by a net transfusion over tiny anastomoses and causing anemia in one twin and polycythemia in the other one, potentially with hemodynamic changes (Gucciardo et al., 2010, Slaghekke et al., 2010). TAPS can arise spontaneously, yet it is better known as a complication of incomplete laser coagulation with missed small communications. Laser can be used to dichorionize the placenta in case of TAPS and also in case of selective intra-uterine growth restriction (sIUGR), yet this has not yet been clinically proven to be of benefit (Gratacos et al., 2008, Chalouhi et al., 2013). Herein we will only focus on laser for TTTS.

Until today TTTS is still the most important cause of death and disability in MC twins (Ortibus et al., 2009). Indeed, untreated mid-trimester TTTS has a 95% mortality, either by miscarriage or preterm delivery or in utero fetal death. As such, surgery of the placenta is imperative to treat this condition. Until the late 1990’s serial amnioreduction was the standard of care, alleviating the polyhydramnios and improving to some extent the placental perfusion. Amnioreduction decreased mortality to 50%, yet about 70% of survivors suffered neurologic morbidity (Senat et al., 2004). A more causative approach is to ablate all inter-twin anastomoses. Surgical ablation is performed under sono-endoscopic control using laser energy with a specific absorbance spectrum for haemoglobin (Hb) (Klaritsch et al., 2009). The ultimate goal of the operation is to dichorionise the placenta to arrest the inter-twin transfusion.

Special instruments were developed in the 1990s to perform this operation percutaneously. A range of fiber endoscope diameters are available (1.0- 2.0 mm), either housed or integrated into straight or curved sheaths. One or more operative channels allow for laser fiber insertion and irrigation of fluid. The sheath will determine the ultimate diameter of the cannula to be used for amniotic sac entry. Diameters of instruments vary according to gestational age. An overview of the material typically used can be found in Table I. We occasionally use a larger diameter, 30° 2.0 mm rod lens HOPKINS ® endoscope (26120BA, Karl Storz GmbH, Tuttlingen, Germany) when optimal visualization is required. Visualisation can be impaired due to cloudy, dense or hemorrhagic amniotic fluid. This rigid scope does not have a working channel, yet it allows more light and, as a rod lens system, a clear image without the typical honey comb pattern of fibre endoscopes.

**Table I:** — Frequently used endoscopes for minimal invasive fetal surgery.

Outer diameter (mm)	Working length (cm)	Angle of view	Type	Flexibility	Angle	Additional details	Company	Reference Nr.
1.0	20.0	0°	Fibre	Semi-rigid	70°	Deported eyepiece	Storz	11510 A
1.3	30.6 0°	0°	Fibre	Semi-rigid	90°	Deported eyepiece	Storz	11540 AA
2.0	30.0	0°	Fibre	Semi-rigid	95°	Deported eyepiece	Storz	11630 AA
2.0	26.0	30°	Rod-lens	Rigid	-	-	Storz	26008 BUA
3.3	30.0	0°	Fibre	Rigid		Deported eyepiece	Storz	11506 AAK
3.3	30.0	0°	Fibre	Rigid		Deported eyepiece	Storz	11508 AAK

## Materials and methods

### Set-up

The operator needs to be familiar with both ultrasound and endoscopic guided procedures. Ultrasound evaluation of fetal and placental position and cord insertion will determine the ideal position for insertion of the fetoscope. Ideally the scope is inserted to obtain a 90° angle to the vascular equator. Obviously, this location is not known for certain prior to surgery as it cannot be identified by the current imaging tools. In practice one presumes it to run perpendicular to the line connecting the two cord insertions. The entry site should provide a maximum overview over the placenta, in particular the presumed equator. The entry point is further determined by physical limitations, such as relevant maternal structures like bowel or vessels. The latter can be avoided by using Doppler ultrasound, identifying uterine or broad ligament vessels, uterine wall vasculature, placental and cord vessels and other fetal structures. The entry is usually chosen in the sac of the recipient to avoid septostomy. Some operators will enter the donor sac in specific cases. When this is done inadvertently at first access, it creates septostomy with its own complications. It is strongly recommended not to enter through the placenta, however that is not always avoidable. Transplacental insertion does cause bleeding, impaired vision, yet also is associated with poorer results (Stirnemann et al., 2013). To obtain optimal visualization and laser firing angle, the patient should be positioned so that an ideal entry site can be obtained. For example, in case the equator is estimated to be vertical anterior, the patient is positioned completely on her side for a maximal lateral entry. In case of a horizontal or transverse equator the operator can stand in between the legs. The screens should be positioned opposite the operators. Ideally, both imaging modalities are visualized.

### Entry

The procedure is performed under strict aseptic conditions. We, in fact, perform this procedure in a full scale operating theatre. The skin is disinfected and covered with sterile drapes. The point of entry is anesthetized with local anesthesia, its depth being controlled under ultrasound control (video 1) — to see all the videos please refer to the QR at the end of this article.

**Figure 3:**
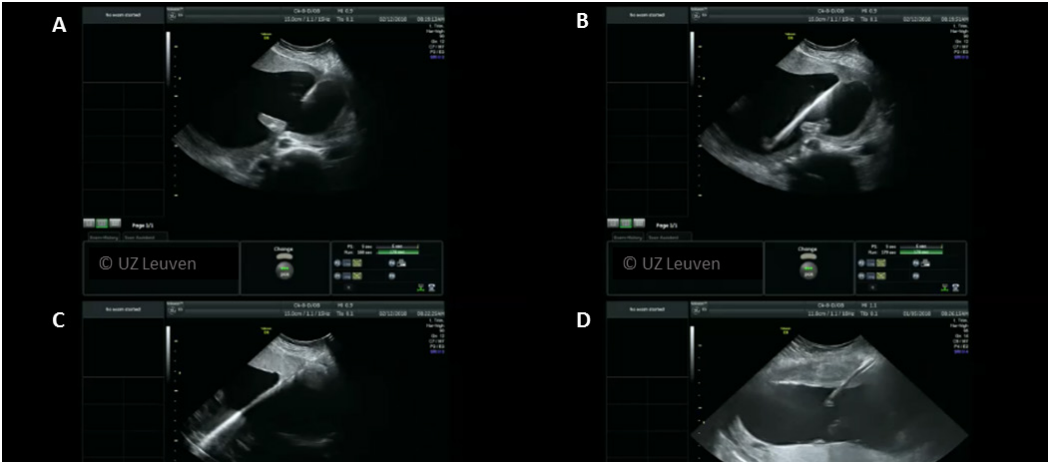
Trocar entry with Seldinger technique. A) A 18G needle is inserted in the uterus. B) The guidewire is advanced through the needle. C) The needle is removed and the cannnula is advanced over the guidewire. D) The guidewire is withdrawn to leave only the cannula as entry to the uterus. Reproduced with permission and copyright: UZ Leuven, Belgium.

In addition to local anesthesia, intravenous sedatives (pethidine, remifentanil, propofol, midazolam) or tocolytics (atosiban, nifedipine) are administered in some centres, depending on the case and gestational age. A small incision is made to allow smoother trocar introduction. The trocar is pyramidal shaped and housed in a thin walled flexible cannula, originally designed for vascular access (Check-Flo Performer ® Introducer Set, Cook Medical Inc., Bloomington, IN, USA). This accommodates curved endoscopes. The diameter of the cannula is dependent on the instrument used. The trocar and cannula are inserted under ultrasound guidance into the large amniotic sac. Others prefer to use the Seldinger-technique (Deprest et al., 1998), as used for vascular access (video 2). In that case, first, a needle is introduced in the amniotic cavity, through which a guide wire is advanced. The needle is removed, following which a dilator and cannula is advanced under vision. Once inside the cavity, the dilator and guidewire are withdrawn (Figure 3, video 3). This technique might be more useful in the absence of polyhydramnios, such as sIUGR and TAPS, but preference is mainly operator dependent. There is at present no evidence that outcomes of one or the other entry technique are better (Petersen et al., 2016).

**Figure 4:**
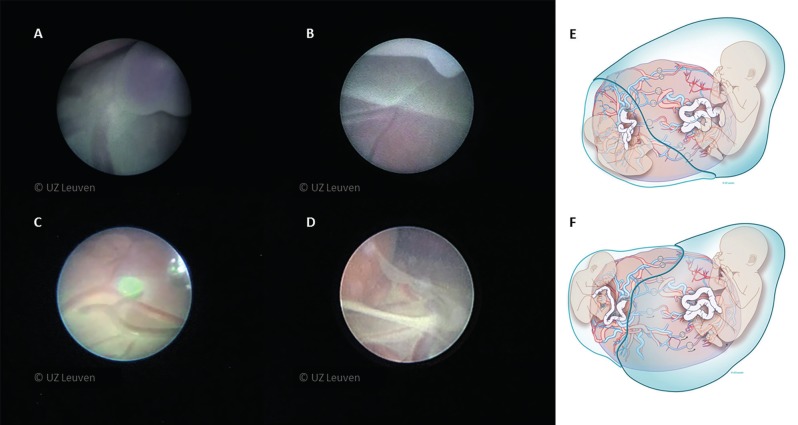
Fetoscopic views of different landmarks used for mapping of the placenta. A) umbilical cord ; B) the membrane appears as a white line, donor vessels can be seen leaving under the membrane ; C) veins have a more typical red (oxygenated) color and arteries are darker (deoxygenated blood) ; D) The donor can be seen stuck under the membranes. E and F) schematic drawings how the stuck twin can relate to the vascular equator. Drawing Myrthe Boymans, all images reproduced with permission and copyright: UZ Leuven, Belgium.

The fetoscope is advanced through the cannula to overlook the surgical field. To assist with this, warm lactated Ringer’s solution can be infused either through the working channel or cannula. This may improve visibility, create more working space and will eventually clear debris from the laser fiber when coagulating. Firstly, the landmarks need to be identified, i.e. the umbilical cord insertions, the inter-twin membrane insertion and the vessels connecting the two fetuses (Figure 4, video 4).

The membrane appears as a white line. Veins and arteries are easily distinguished. In utero veins have a more typical red (oxygenated) color while arteries are darker (deoxygenated blood). Furthermore, arteries cross over the veins, thus the placental borders, cord insertions and the vessels arising from them need to be carefully mapped. This may be hindered by fetal structures, or the stuck twin. In that case, donor vessels can be seen leaving from under the donor without returning and rather going in the direction of the equator and the recipient. When one cannot determine whether a vessel is anastomosing or not, we advise coagulating it.

### Selective laser

**Figure 5:**
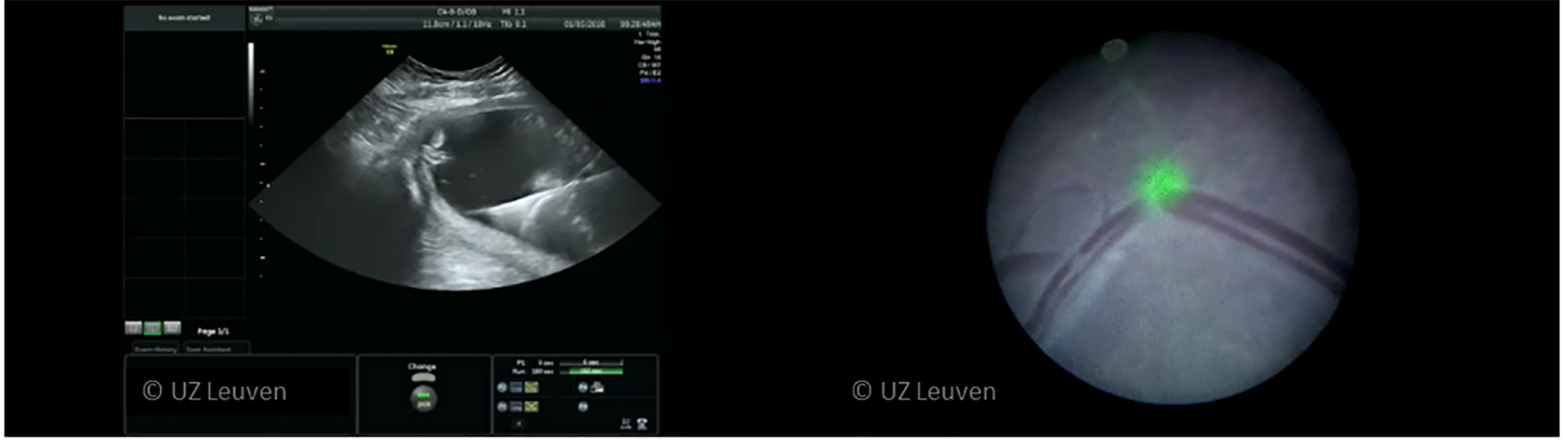
Typical view with ultrasound (left) and fetoscopically (right) during active lasering. Reproduced with permission and copyright: UZ Leuven, Belgium.

Laser coagulation of the anastomoses is performed from approximatively 1cm distance (Figure 5). Typical lasers used in this type of surgery are neodymium: yttrium–aluminium–garnet (Nd:YAG; wavelength 1064 nm, reported power requirements 50–100 W, e.g. MedilasFibertom 8100; Dornier MedTech, Wessling, Germany) or diode (940 nm, 20–60 W, e.g. Medilas D Multibeam; Dornier Med- Tech) (Akkermans et al., 2015a). Laser energy should be adapted to the working distance, type and size of the vessels. Contact between laser fiber and tissue should be avoided at all costs. The laser can also be fired through the membrane in case vessels behind the membrane need to be coagulated. Bursts of energy are fired over 3 to 4 seconds to coagulate the vessel. Vessels and amnion will turn white as a sign of coagulation. In case of larger vessels (>3mm), multiple shots along the course of the vessel may be necessary to progressively obtain narrowing and finally completely obliterating it (video 5). This should be done cautiously at low energy levels as it risks to perforate and cause fetal bleeding which might be lethal on the short term. On the other hand slightly higher laser energy can be more energy efficient and cause less placental damage (Akkermans et al., 2017a, b).

### Sequential laser

From a surgical viewpoint, it is important that AA and VVs are direct communications between arteries and veins respectively on the surface of the placenta (Figure 1). AAs and VVs are therefore called superficial anastomoses. In contrast, in an AV anastomosis typically the artery of one twin dips into the placenta a few millimeters from a vein of the other twin (Figure 2; inset). AVs are therefore called deep anastomoses. When uncompensated, the blood supplied by the artery of one fetus will then be entirely drained to a vein from the other fetus. From a surgical point of view, it is important to correctly identify the different types of anastomoses to allow grouped coagulation of AV, AA and VV, known as sequential lasering (Quintero et al. 2007). In this technique first AV (donor to recipient) are coagulated, followed by VA (recipient to donor). Sequential lasering has been associated with improved survival rates in several non-randomised studies (Quintero et al., 2007, Akkermans et al., 2015a,b,c).

### Solomon technique

**Figure 6:**
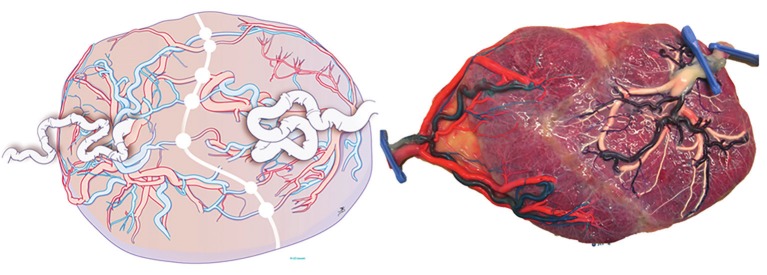
Solomon-technique: after coagulating the anastomoses, coagulation points are connected by a continuous line from one placental border to the other for complete coagulation of all small vessels that may have been missed initially. This should lead to functional “dichorionization”. Drawing Myrthe Boymans, all images reproduced with permission and copyright: UZ Leuven, Belgium.

Once all anastomoses are coagulated, it is now advised that coagulation points are connected by a line from one placental border to the other for complete coagulation of all small vessels that may have been missed initially (“Solomon” technique) (Figure 6, video 6). The superiority of this technique was demonstrated in a randomized trial comparing it to conventional lasering without the “line” (Slaghekke et al., 2014a,b). Some recent observational reports suggest that lining of the placenta might increase the risk for placental abruption or PPROM (Lanna et al., 2017, Stirnemann et al., 2018), although this was initially not reported in the Solomon trial. This could be explained by the longer time of the procedure and possibly wider manipulation of the cannula and scope.

Before ending the procedure, a final check can be performed to optimally visualize the coagulation trajectory using the rod lens-scope, which has a wider lens because it does not have an operative channel (video 7). Amniotic fluid is then drained till normal levels. The cannula is removed under ultrasound guidance.

### Anterior placenta

**Figure 7:**
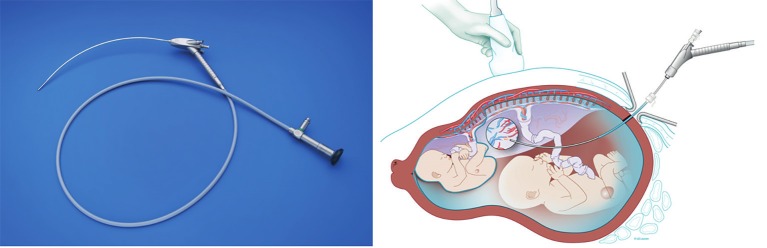
For an anterior placenta a ‘curved scope can be used to achieve a more perpendicular view. Drawing Myrthe Boymans, reproduced with permission and copyright: UZ Leuven, Belgium. Photo: reproduced with permission from Karl Storz, Tuttlingen, Germany.

In case of an anterior placenta, the straight scope can be switched for a curved model which allows more complete vision of the placenta and a better angle between the laser and the placenta for optimal energy impact (Figure 7). By positioning the patient in extreme lateral tilt the maximum lateral insertion point can be chosen to further optimize the angle. An alternative is to advance the cannula till it makes contact with the placenta, which tumbles the latter in front of it. Then the laser can be fired from within the cannula, yet at a lower energy level (Klaritsch et al., 2009). Other options have been tried to overcome this problem, including a mini laparotomy for fundal access to the uterus or laparoscopic assistance (Deprest et al., 1998, Papanna et al., 2010). Quintero et al described the use of a thicker laser fiber with side- firing capabilities (Quintero et al., 2001). This requires a second working channel as the thicker fiber does not match the working channel. Yet another option is to use a 30° (rod lens) scope with an Albarran deflecting mechanism which enables the operator to deflect the laser fiber towards the target (Huber et al., 2008). This scope is rather wide and relatively short.

### Follow-up

Patients are examined by ultrasound the day after the procedure to assess possible complications such as fetal demise, the occurrence of inadvertent septostomy or membrane separation and cervical length. In most countries monochorionic twins are followed up by two-weekly ultrasound. Postoperative surveillance even includes weekly ultrasound initially (Khalil et al., 2016b). Apart from surgical complications and recurrence, fetal growth, brain, cardiac and limb development are to be followed up. Given that survivor after laser surgery are at inceased risk for neurological damage, some centers perform postoperative MRI, yet that is investigational (Weisz et al., 2014). If patients do not deliver spontaneous, we advocate delivery around 35 weeks after a course of steroids as the risk for placental dysfunction increases afterwards (Stirnemann et al., 2012).

## Results

### Short term fetal outcome

The most important early complication is in utero fetal demise of one or both twins (45% and 12% respectively (Akkermans et al., 2015a,b). Early procedures (<17 weeks) have poorer outcomes than later ones, mainly by higher PPROM rates (Stirnemann et al., 2018). TAPS is caused by missed small anastomoses, up to 3% after dichorionization (Lewi et al., 2006; Slaghekke et al., 2014a). More uncommon is persisting or recurrent TTTS , which occurs in about 1%, e.g. due to technical difficulties with missed large anastomoses (Lewi et al., 2006; Slaghekke et al., 2014b). Management will depend on gestational age and technical aspects. A second laser is possible but can be complicated by hemorrhagic amniotic fluid caused by the previous intervention.

### Short term maternal outcome

Immediate postoperative maternal complications include peritoneal irritation due to amniotic fluid leakage and/or blood in the abdominal cavity. Although rare, hemorrhage, infection and placental abruption have been described. The clinically most relevant postoperative problems are preterm labor and delivery and membrane rupture. Iatrogenic PPROM is described in 27% of cases, potentially higher when using the Solomon technique (Beck et al., 2012; Akkermans et al. 2017a).

### Long term fetal outcome

There is level I evidence that primary laser coagulation improves survival. The Eurofoetus group showed that survival at 6 months in at least one twin was significantly higher and average delivery was later. In a recent systematic review including 34 studies, laser compared to drainage, was associated with an increased survival of both twins from 35 to 65% and survival of at least one twin from 70 to 88% (Akkermans et al., 2015a). In the Eurofoetus trial, the occurrence of cystic periventricular leukomalacia was lower after laser than after amniodrainage (Senat et al., 2004). The neurologic benefit persisted in the long term (Salomon et al., 2010). In a recent systematic review, the long term neurodevelopmental impairment was 10% after laser (van Klink et al., 2016).

For stage I the optimal management remains controversial (Khalil et al., 2016a). There is currently an ongoing trial comparing immediate laser coagulation with expectant management followed by laser coagulation if necessary.

Potential complications include thermal injury. This may happen e.g. due to unexpected fetal movements, reportedly with minor or no consequences (Soriano- Ramos et al., 2019). However, this happens infrequently and heals easily and completely. This should be differentiated from aplasia cutis congenita, in which lesions are symmetrical (Mugarab-Samedi et al., 2017). Another rare complication is vascular limb occlusion. This complication can vary in severity from superficial skin damage to total limb amputation and is mostly seen in lower extremities of the recipient. However, the underlying mechanisms remain uncertain. It seems to be more likely a complication of TTTS than of laser therapy as it has been described after amnioreduction and in untreated MC pregnancies (Schrey et al., 2012).

### Long term maternal complications

Laser therapy seems to be safe for the mother with no impact on subsequent fertility and pregnancies, or gynaecologic complications in the long term (Vergote et al., 2018). However, women who underwent laser coagulation were more likely to report relevant psychological symptoms, especially in the group with demise of one or both twins. This underscores the need for long term support of these patients.

## Conclusion

Laser surgery is the standard of care for TTTS because it is safe and it significantly improves survival rates and neurologic outcome compared to amniodrainage. Survival rates can still improve and membrane rupture rates should be reduced. Research is ongoing into thinner and flexible instruments, access port sealing devices, instrument stabilization or robotic assistance, to name a few.

## Funding

JD is partly funded by the Great Ormond Street Hospital Charity Fund. LVDV and JvdM is supported by the Erasmus+ Programme of the European Commission (2013-0040). LL is funded by the FWO (Fonds voor Wetenschappelijk Onderzoek) (1804718N). Our research on novel instrumentation in fetal surgery is supported by an Innovative Engineering for Health award by the Wellcome Trust (WT101957) and the Engineering and Physical Sciences Research Council (ESPRC) (NS/A000027/1).

## Video scan (read QR)

Supplementary video 1: Local anesthesia is injected under ultrasound guidance.

Supplementary video 2: Trocar entry with Seldinger technique. Under ultrasound guidance a 18G needle is inserted in the uterus. The guidewire is advanced through the needle. Then the needle is removed and the cannula advanced over the guidewire. The guidewire is then with- drawn to leave only the cannula as entry port to the uterus

Supplementary video 3: These are the landmarks to be found on the placenta: umbilical cord insertions, the intertwin membrane insertion and the vessels connecting the two fetuses.

Supplementary video 4: Small vessels can be coagulated using short bursts of energy. The vessels and amnion will turn white as the proteins denaturate, a sign of coagu- lation. In case of larger vessels (>3mm), multiple shots along the course of the vessel might help to progressively obtain narrowing and finally completely obliterate it.

Supplementary video 5: Solomon-technique: after coagulating the anastomoses, these points will be connected by a continuous line from one placental margin to the other for complete coagulation of all small vessels that may have been missed initially hence cause functional dichorionization.

Supplementary video 6: Before ending the procedure a final check can be performed to visualize the coagulation trajectory.

**Figure:**